# Late functional improvement and 5-year poststroke outcomes: a population-based cohort study

**DOI:** 10.1136/jnnp-2019-322365

**Published:** 2020-06-23

**Authors:** Aravind Ganesh, Ramon Luengo-Fernandez, Peter Malcolm Rothwell

**Affiliations:** 1 Wolfson Centre for Prevention of Stroke and Dementia, Nuffield Department of Clinical Neurosciences, University of Oxford, Oxford, UK; 2 Department of Clinical Neurosciences, University of Calgary, Calgary, Alberta, Canada

## Abstract

**Background:**

Late functional improvement between 3 and 12 months poststroke occurs in about one in four patients with ischaemic stroke, more commonly in lacunar strokes. It is unknown whether this late improvement is associated with better long-term clinical or health economic outcomes.

**Methods:**

In a prospective, population-based cohort of 1-year ischaemic stroke survivors (Oxford Vascular Study; 2002–2014), we examined changes in functional status (modified Rankin Scale (mRS), Rivermead Mobility Index (RMI), Barthel Index (BI)) from 3 to 12 months poststroke. We used Cox regressions adjusted for age, sex, 3-month disability and stroke subtype (lacunar vs non-lacunar) to examine the association of late improvement (by ≥1 mRS grades, ≥1 RMI points and/or ≥2 BI points between 3 and 12 months) with 5-year mortality and institutionalisation. We used similarly adjusted generalised linear models to examine association with 5-year healthcare/social-care costs.

**Results:**

Among 1288 one-year survivors, 1135 (88.1%) had 3-month mRS >0, of whom 319 (28.1%) demonstrated late functional improvement between 3 and 12 months poststroke. Late improvers had lower 5-year mortality (aHR per mRS=0.68, 95% CI 0.51 to 0.91, p=0.009), institutionalisation (aHR 0.48, 0.33 to 0.72, p<0.001) and healthcare/social care costs (margin US$17 524, –24 763 to −10 284, p<0.001). These associations remained on excluding patients with recurrent strokes during follow-up (eg, 5-year mortality/institutionalisation: aHR 0.59, 0.44 to 0.79, p<0.001) and on examining late improvement per RMI and/or BI (eg, 5-year mortality/institutionalisation with RMI/BI: aHR 0.73, 0.58 to 0.92, p=0.008).

**Conclusion:**

Late functional improvement poststroke is associated with lower 5-year mortality, institutionalisation rates and healthcare/social care costs. These findings should motivate patients and clinicians to maximise late recovery in routine practice, and to consider extending access to proven rehabilitative therapies during the first year poststroke.

## Introduction

Functional improvement after neurological lesions like stroke involves neural recovery, through structural and functional plasticity,^1^ and reflects the individual’s physiological and psychosocial adaptation to activities with residual impairments.^2 3^ Improvement after ischaemic stroke was conventionally thought to occur mostly in the first 3 months poststroke, plateauing at about 6 months without additional significant improvement.^4^ However, a few strategies have demonstrated efficacy in promoting recovery well beyond this window. For example, constraint-induced movement therapy (CIMT) has been effective in the 3–9 months poststroke window.^5 6^ This has led to the realisation that exceptions exist and that patients may demonstrate late functional improvement, some even several years poststroke, generating interest in developing further interventions for continued recovery and models to predict potential for late improvement.^7^ In prior analyses of 5-year functional outcomes in the Oxford Vascular Study (OXVASC), we have demonstrated that late functional improvement between 3 and 12 months poststroke occurs in about one in four patients with ischaemic stroke.^8^ We have also shown that such late improvements, as captured by measures like the modified Rankin Scale (mRS), Rivermead Mobility Index (RMI) and the Barthel Index (BI), occur more commonly in patients with lacunar strokes compared with those with non-lacunar ischaemic strokes.^9^

Although late functional improvement may seem intuitively desirable for patients, it is unknown whether such improvement is actually associated with better long-term outcomes. We have previously demonstrated that each increment in 3 months mRS is associated with worse 5-year outcomes and costs,^10^ but the association of late functional improvement with such clinical or health economic outcomes remains to be studied. Whereas cohort-based associations between late improvement and long-term outcomes cannot prove causation or speak to the efficacy of rehabilitative therapies, it is worth noting that insurance policies in countries like the USA often restrict patients with stroke from accessing rehabilitation after hospital discharge.^7^ Even in countries with universal healthcare insurance like the UK and Canada, patients struggle to access rehabilitation services beyond the first few months poststroke.^11^ If late improvement meaningfully improves long-term outcomes like mortality, institutionalisation or healthcare/social care costs, this can further motivate clinicians and patients to maximise the use of late restorative therapies with known efficacy, and may encourage payers to invest in the coverage and development of such therapies. More importantly, in the absence of long-term outcome data, it can be argued that late functional improvement is simply the result of patients better adapting to poststroke deficits over time, rather than neurological recovery. Besides, functional scales like the mRS may fluctuate from one visit to the next due to health or psychosocial factors unrelated to stroke and are subject to inter-rater variability^12^; should apparent late improvement merely represent such fluctuations, one would not expect long-term trajectories to differ considerably between patients with/without such improvement. On the other hand, demonstrating the predictive validity of observed late functional improvement with long-term outcomes will help establish this improvement—regardless of its biological or psychosocial roots—as a clinically meaningful phenomenon in stroke care. Therefore, we examined how late functional improvement between 3 and 12 months poststroke translates into 5-year outcomes.

## Methods

The OXVASC population comprises 92 728 patients registered with about 100 general practitioners(GPs) in 9 practices across Oxfordshire. Study methods have been published.^13^ Recruitment has been ongoing since April 2002. Near-complete ascertainment of suspected stroke/transient ischaemic attack (TIA) cases is achieved using overlapping methods of ‘hot’ and ‘cold’ pursuit, as previously described.^14^ Patients with ischaemic stroke recruited from April 2002 to March 2014 were included. Patients were assessed urgently by study clinicians. Stroke was diagnosed per the WHO definition.^15^ Stroke severity was measured using the National Institutes of Health Stroke Scale (NIHSS). Based on standard investigations, including neuroimaging, vascular imaging, echocardiography and rhythm monitoring, we classified stroke subtypes using Trial of Org 10172 in Acute Stroke Treatment (TOAST) criteria.^16^ Patients received no interventions beyond standard medical management of stroke, with inpatient or community-based rehabilitation decisions left to the discretion of the patients’ attending team.

Patients had face-to-face follow-up with a study nurse/physician in clinic or at home at 1 month, 3 months, 6 months, 1 year and 5 years. At each visit, functional status was assessed using the mRS, RMI and BI. The mRS is a 7-point disability scale ranging from 0 (no symptoms) to 6 (death).^12^ The RMI assesses functional mobility tasks and ranges from 0 (cannot perform any) to 15 (can perform all).^17^ The BI assesses activities of daily living (ADLs) and ranges from 0 (dependent for all) to 20 (independent for all).^18^ These scales are often used as outcome measures in trials of post-stroke restorative therapies^19^; in particular, the mRS and BI have been the first and second-most commonly used functional outcome measures in stroke trials.^20^ Whereas the mRS overlaps the realms of impairment, disability and handicap, the BI offers a purer assessment of disability (ADLs) while the RMI is more geared towards impairment (specifically in mobility). Using these scales allowed us to better capture the full spectrum of the WHO framework of disease.^21^

Raters were trained in mRS assessment using an instructional DVD (Digital Versatile Disc) with written materials produced by the University of Glasgow, previously used in large-scale trials,^12^ and underwent additional training and observation for RMI and BI assessments. Prestroke mRS and BI were determined at enrolment. At follow-up, patients/carers were asked about living arrangements. In addition, patients’ GP/hospital records were reviewed to identify if/when they were institutionalised. Institutionalisation was defined as admission into a nursing or residential care home, excluding temporary post-acute care and in-hospital rehabilitation stays.

Patients who moved out of study area received telephone follow-up. Additional information was obtained from carers in patients with significant speech/cognitive impairment. Recurrent vascular events were identified by daily OXVASC ascertainment, follow-up interviews and review of GP/hospital diagnostic codes. Deaths were recorded from death certificates, coroners’ reports and the National Health Service (NHS) Central Register. Health and social care resource use was obtained from the date of the first stroke in study period (‘index’ stroke) until 5 years poststroke, as reported previously.^22^ Briefly, information was obtained from face-to-face follow-up, GP and hospital records regarding any emergency visit/transport, outpatient-care visit, day case or hospitalisation, including hospital-based rehabilitation (with length of stay) and community-based rehabilitation (physiotherapy, speech/language, occupational therapy). We estimated institutionalised days as the difference between either date of 5-year follow-up or death, whichever was earliest and date of admission into the institution. Hospital resource use was valued using unit costs from the NHS schedule of reference costs.^23^ Institutionalisation was costed as the cost per week in a private nursing home, £795(US$1145) in 2016.^24^ All costs were presented in 2016 prices and converted from UK pounds sterling(£) to US dollars($) using the 2016 rate of purchasing power parities (US$=£0.694, http://stats.oecd.org/).

### Standard protocol approvals, registrations and patient Consents

Informed consent was obtained from patients whenever possible; otherwise, assent was obtained from caregivers if patients were unable to consent.

### Statistical analyses

Only patients surviving ≥12 months after their index stroke were included. Analyses were censored at 1 May 2017.

Any drop in mRS is meaningful, as long-term mortality and dependency rise with each scale increment,^10^ so patients were deemed to show late functional improvement if the score decreased by ≥1 grades between 3 and 12 months. Patients who improved between 3 months and 6 months but then declined between 6 months and 1 year were still considered to have demonstrated late improvement. We compared mortality and institutionalisation rates in those with and without late improvement; since patients with lacunar strokes have lower long-term disability and mortality and are more likely to show late improvement, we also examined non-lacunar strokes separately to ensure that differences were not driven by differing proportions of lacunar strokes.^9^ Cox regressions were used to model the association of late improvement with 5 years mortality and institutionalisation, adjusted for age, sex, 3 months mRS and stroke subtype (lacunar vs non-lacunar). Patients with 3 months mRS=0 were excluded since they could not show improvement. We examined the association between late improvement and 5 years healthcare/social care costs using similarly adjusted generalised gamma linear models (GLMs), assuming a log identity. To verify that differences were not reflecting non-stroke-related disability, we repeated these regressions progressively excluding patients with recurrent vascular events and prestroke mRS >2, and adjusting for comorbidities that differed significantly between those with and without late improvement. Strictly speaking, however, improvement from mRS=1 (symptoms without disability) to mRS=0 (no symptoms) is not true functional improvement. To focus on patients with mild-to-moderate disability who might be best suited for rehabilitation therapies, we therefore repeated the analysis using only patients with 3 months mRS 2–4.

Even so, there are limitations to the degree of functional improvement that can be captured by the mRS, given there are only six possible states (excluding mRS=6/death). Therefore, we validated our findings by repeating these analyses with the RMI and BI, which have 15 and 20 points, respectively. The RMI’s minimal clinically important difference (MCID) is not established, but test/retest studies suggest that increases by ≥1 points are reliable, so this was deemed indicative of functional improvement.^25^ The BI’s MCID is 1.85 points, so increases by ≥2 points were deemed indicative of improvement.^18^ Patients with 3 months RMI=15 and BI=20 were excluded as they could not show improvement.

In additional sensitivity analyses, we repeated the Cox regressions and GLMs separately for late improvement between 3 and 6 months and between 6 and 12 months poststroke, to see whether associations with 5-year outcomes differed based on the timing of the improvement. We also repeated the Cox regressions and GLMs with late improvement (per mRS/BI/RMI) coded as numerical variables to reflect the full spectrum of improvement. These analyses were also adjusted for age, sex, TOAST subtype, 3-month disability and recurrent events during the follow-up.

Statistical analyses used STATA V.13.1. Trends in continuous/ordinal data were compared using non-parametric Wilcoxon rank-sum tests corrected for ties. Dichotomous variables were compared using X^2^ tests. Significance was set at p<0.050.

Requests for access to the data used in this paper will be considered by the corresponding author.

## Results

**Figure 1:**
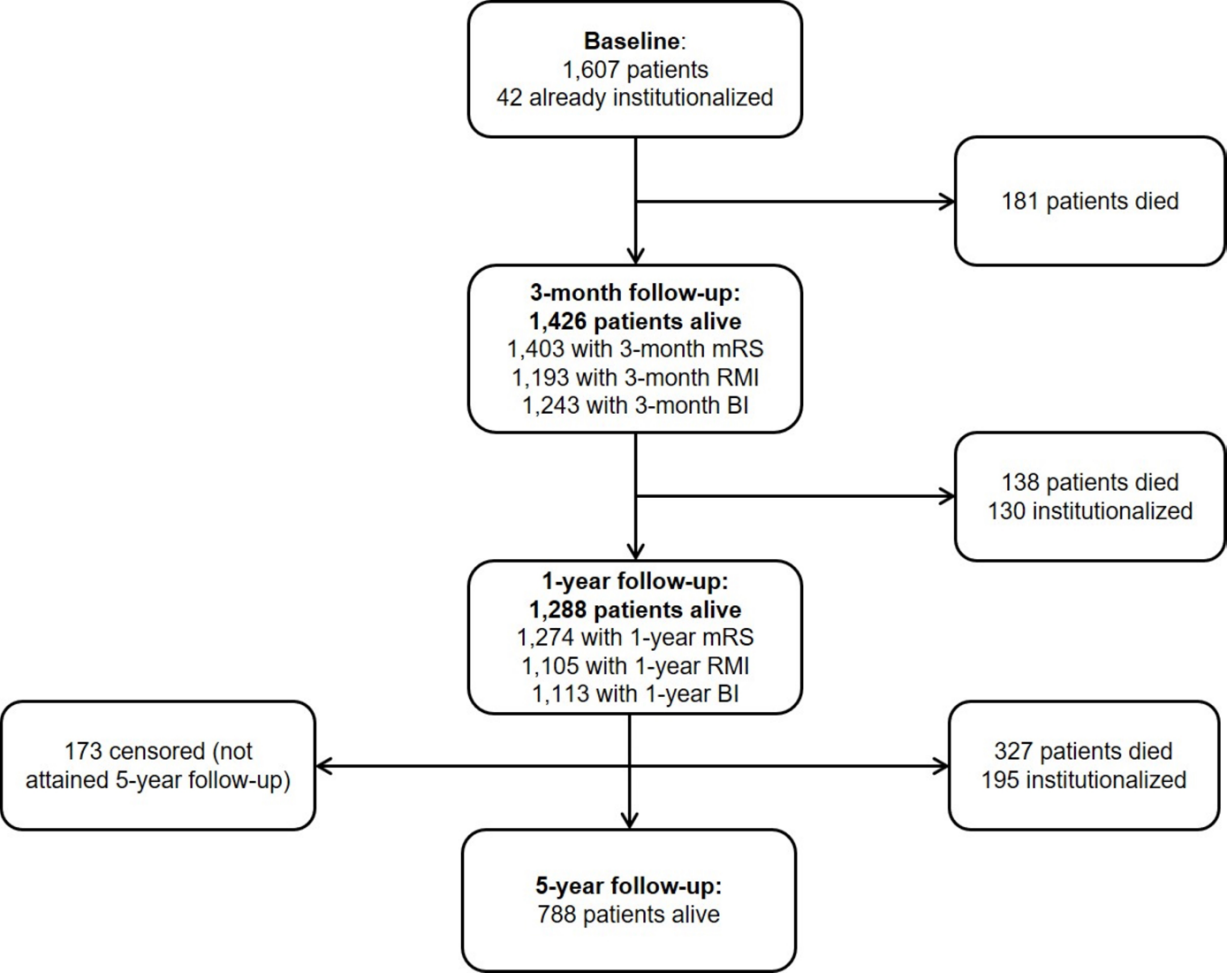
Flow chart illustrating patients with ischaemic stroke who were alive at baseline and at 3-month, 1-year and 5-year follow-up assessments. A number of patients with available follow-up data at each time point are also indicated, as well as the number who were newly admitted to a nursing or residential care home. BI, Barthel Index; mRS, modified Rankin Scale; RMI, Rivermead Mobility Index.

Among 1607 patients with ischaemic stroke, 181 patients died within 3 months and 138 others died within 1 year (figure 1). Both 3-month and 1-year functional status were available for ≥1 scale (mRS/RMI/BI) for 1271 (98.7%) of 1288 1 year survivors. One hundred and seventy-three patients (13.4%) had not yet reached 5-year follow-up, but all had at least 3-year follow-up data; therefore, both functional status and follow-up mortality/institutionalisation/cost data were available for 98.7% of the cohort. A total of 1135 one-year survivors had 3-month mRS >0, of whom 319 (28.1%) demonstrated late improvement per the mRS between 3 and 12 months poststroke. A total of 707 one-year survivors had 3-month RMI <15; 351 (49.7%) showed late RMI improvement. A total of 451 one-year survivors had 3-month BI <20; 158 (35.0%) showed late BI improvement. Seventeen patients who improved on the mRS between 3 and 6 months but then declined between 6 and 12 months were still considered to have shown late improvement; corresponding numbers for RMI and BI were 35 and 22 patients, respectively.

Patients demonstrating late improvement per the mRS were younger, more often male, less likely to have prior angina or TIA, premorbid mRS >2 or premorbid BI <20, and more likely to have lacunar strokes (table 1). They did not differ in initial stroke severity (NIHSS: median 2, IQR 1–4); receipt of thrombolysis, inpatient or outpatient rehabilitation (including the timing of last rehabilitation sessions); history of MI, stroke or other comorbidities (eg, cancer); poststroke depression; or recurrent vascular events over 5-year follow-up. Characteristics of those who did and did not demonstrate late improvement per RMI and BI are shown in (online supplementary eTables 1,2). Patients demonstrating late improvement per mRS had higher (ie, worse) 3-month mRS scores than those who did not improve (median mRS.2, IQR.2–3 for improvement vs 2, 1–3 for no improvement, p<0.0001) and those improving per RMI/BI had lower (ie, worse) 3-month RMI/BI scores than those who did not improve (median RMI.11, IQR.7–13 vs 12, 8–14 for no improvement, p=0.0055; median BI.13, IQR.8–17 vs 18, 13–9, p<0.0001). The majority of patients with late improvement improved between 3 and 6 months (eg, 209 (65.5%) for mRS), but a considerable proportion also improved between 6 and 12 months (124 (38.9%) for mRS), with a small number showing improvement in both time periods (14 (4.4%) for mRS; RMI/BI data in online supplementary eTables 1,2). Patients with late improvement per mRS and BI were more likely to have accessed rehabilitation services specifically between 3 and 12 months poststroke (eg, BI: 22.2% of improvers vs 11.6% of non-improvers, p=0.003) although this difference was attenuated on adjusting for age/sex/subtype/3 month disability (eg, adjusted odds ratio (aOR), BI: 1.35, 0.76–2.40, p=0.31).

10.1136/jnnp-2019-322365.supp1 Supplementary data

**Table 1:** Characteristics of 1-year survivors of ischaemic stroke who did and did not demonstrate functional improvement per the modified Rankin Scale between 3 months and 1-year poststroke

Characteristic	Late improvement (n=319)	No late improvement (n=816)	P value	P value (age adjusted)
**Age, mean (**SD)	70.1 (12.3)	73.9 (12.7)	<0.0001*	
**Sex: male (%**)	186 (58.3)	404 (49.5)	0.008*	0.056
**Previous history (%)**				
Myocardial infarction	35 (11.0)	113 (13.9)	0.20	0.36
Angina	41 (12.9)	157 (19.2)	0.011*	0.043*
Atrial fibrillation	51 (16.0)	146 (17.9)	0.45	0.98
Hypertension	195 (61.1)	523 (64.1)	0.35	0.97
Dyslipidaemia	102 (32.0)	285 (34.9)	0.35	0.34
Diabetes	46 (14.4)	122 (15.0)	0.82	0.77
Peripheral vascular disease	21 (6.6)	64 (7.8)	0.47	0.55
Stroke	26 (8.2)	96 (11.8)	0.077	0.20
Transient ischaemic attack	34 (10.7)	136 (16.7)	0.011*	0.030*
Smoking	197 (61.8)	465 (57.0)	0.14	0.34
Heart failure	18 (5.6)	70 (8.6)	0.096	0.20
Valvular heart disease	29 (9.1)	81 (9.9)	0.67	0.93
Cancer	48 (15.1)	117 (14.3)	0.76	0.29
Prestroke mRS >2	22 (6.9)	150 (18.4)	<0.001*	0.001*
Prestroke BI <20	31 (9.9)	188 (24.2)	<0.001*	<0.001*
**Initial NIHSS**, mean (SD)	3.8 (4.8)	3.6 (4.7)	0.59	0.33
**TOAST subtype (%**)				
Lacunar (small vessel disease)	75 (23.5)	138 (16.9)	0.010*	0.031*
Cardioembolism	73 (25.4)	182 (24.6)	0.79	0.51
Large artery atherosclerosis	32 (11.1)	79 (10.7)	0.84	0.87
Undetermined	76 (26.4)	224 (30.2)	0.22	0.12
Unknown	18 (6.3)	79 (10.7)	0.030*	0.093
**Received thrombolysis (%**)	7 (2.4)	9 (1.2)	0.17	0.16
**Received in-hospital rehabilitation (%**)	108 (33.9)	233 (28.9)	0.08	0.19
Length of stay for rehabilitation, median days (IQR)	34.5 (13.5–73)	35 (16–82)	0.39	0.93
Timing of in-hospital rehab completion, median days poststroke (IQR)	24 (9–70)	27 (7–85)	0.85	0.72
**Received community-based rehabilitation (%**)	53 (16.6)	125 (15.3)	0.59	0.89
No of sessions, median (IQR)	4 (1–10)	2 (1–6)	0.053	0.23
Timing of rehabilitation completion, median days poststroke (IQR)	869 (229–1618)	671 (228–1255)	0.53	0.46
**Received hospital-based or community-based rehab between 3 and 12 months (%**)	42 (13.2)	72 (8.8)	0.029*	0.024*
**3 month mRS**, median (IQR)	2 (2–3)	2 (1–3)	<0.0001*	<0.0001*
mRS=1 (%)	66 (20.7)	350 (42.9)		
mRS=2 (%)	127 (39.8)	169 (20.7)		
mRS=3 (%)	58 (18.2)	165 (20.2)		
mRS=4 (%)	52 (16.3)	87 (10.7)		
mRS=5 (%)	16 (5.0)	45 (5.5)		
**Late mRS improvement timing (%)†**				
3–6 months poststroke	209 (65.5)	0	<0.0001*	
6–12 months poststroke	124 (38.9)	0	<0.0001*	
**Recurrent**.**stroke** within 5 years (%)	52 (16.3)	118 (14.5)	0.44	0.27
**Any recurrent vascular event** within 5 years (%)	86 (27.0)	213 (26.1)	0.77	0.53
**Poststroke depression** (%)	81 (25.4)	219 (26.8)	0.62	0.55

We compared ordinal/continuous variables using the Wilcoxon rank-sum (Mann-Whitney U) and dichotomous variables using χ^2^ tests.

Only patients with 3-month MRS >0 (capable of showing further improvement) are included.

*Indicated the significant differences (p<0.05).

†14 patients showed improvement on the mRS both between 3 and 6 months and further between 6 and 12 months.

BI, Barthel Index; mRS, modified Rankin Scale; NIHSS, National Institutes of Health Stroke Scale; TOAST, Trial of Org 10172 in Acute Stroke Treatment.

**Figure 2:**
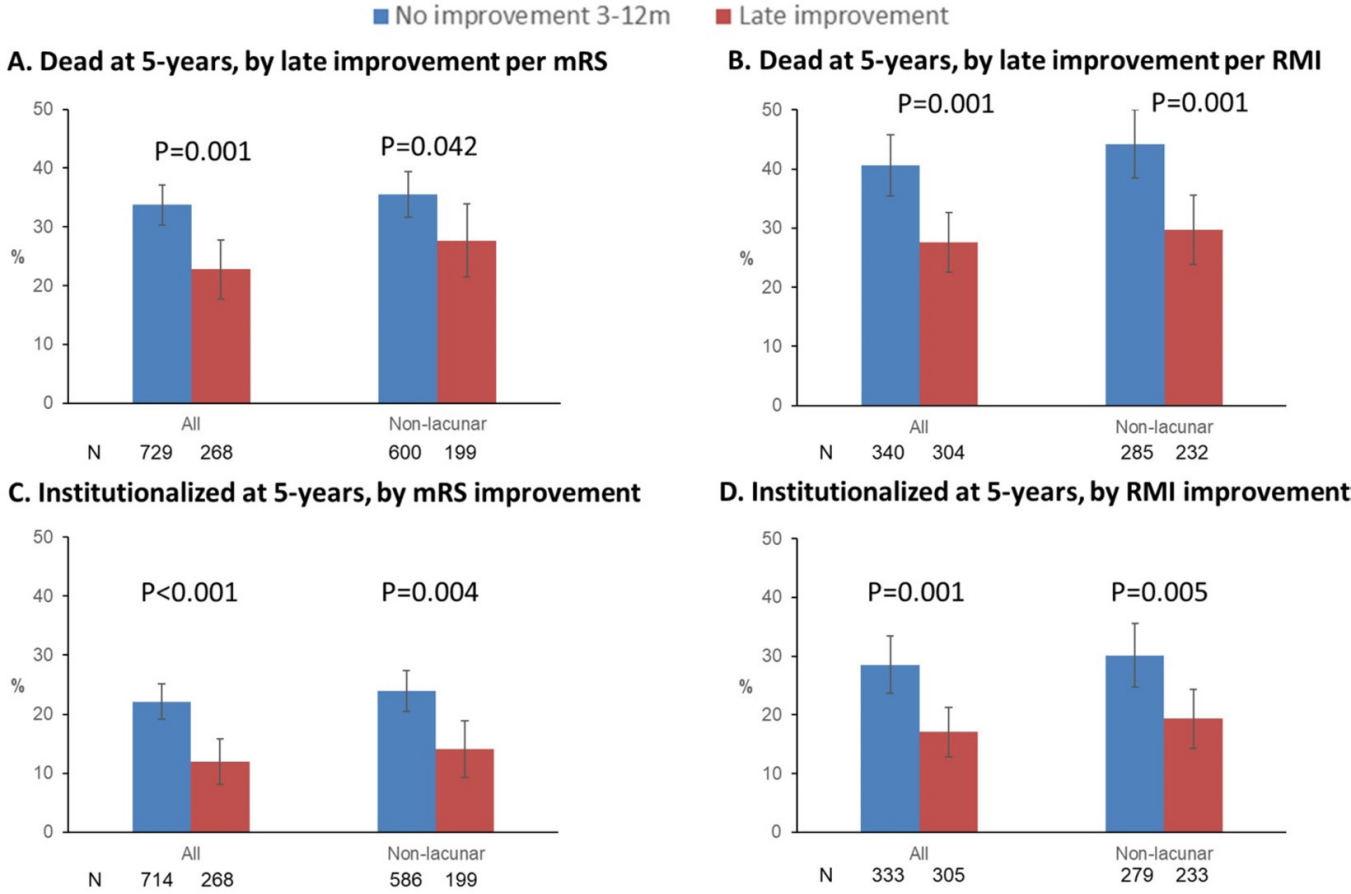
Proportion of 1-year survivors with 3-month mRS >0 (A, C) or 3-month RMI <15 (B, D), grouped by whether they improved between 3 and 12 months per the MRS (A, C) or RMI (B, D), who were (A, B) dead or (C, D) newly admitted to a nursing or residential care home by 5 years. P values are from X^2^ tests. Bars represent 95% CIs. N is the number of patients contributing to the denominator in each bar. Only patients with full 5-year follow-up (or who met the outcome in the interim) were included. mRS, modified Rankin Scale; RMI, Rivermead Mobility Index.

One-year survivors who demonstrated late improvement between 3 and 12 months per mRS had lower 5-year mortality, both in the overall cohort and when non-lacunar strokes were examined separately (figure 2A). Late improvement per mRS was also associated with lower mortality on Cox regressions (adjusted hazard ratio (aHR) for mortality: 0.68, 95% CI 0.51 to 0.91, p=0.009, online supplementary eTable 3). Similar results were seen using RMI and BI (figure 2B, aHR for mortality with RMI improvement: 0.64, 0.49 to 0.85, p=0.002, online supplementary eTable 3). These associations remained significant on excluding patients with recurrent strokes during the follow-up (eg, aHR with mRS/RMI/BI improvement: 0.65, 0.50 to 0.85, p=0.001, online supplementary eTable 4), and on further excluding those with pre-morbid mRS >2 and adjusting for potentially confounding differences in comorbidities like prior angina and TIA (aHR with mRS/RMI/BI improvement: 0.68, 0.51 to 0.90, p=0.006, online supplementary eTable 5).

Similarly, 1-year survivors with late mRS improvement between 3 and 12 months had lower 1-year institutionalisation (aHR: 0.32, 0.16 to 0.63, p=0.001) and 5-year institutionalisation (aHR: 0.48, 0.33 to 0.72, p<0.001), including among those with non-lacunar strokes, with similar results for RMI and BI (figure 2C, D, online supplementary eTables 6,7). Associations remained significant on excluding patients with recurrent strokes (eg, aHR at 5 years with RMI: 0.48, 0.32 to 0.71, p<0.001, online supplementary eTable 8) and premorbid disability, further adjusting for other confounders (eg, aHR with mRS/RMI/BI improvement: 0.56, 0.37 to 0.85, p=0.006, online supplementary eTable 9).

**Figure 3:**
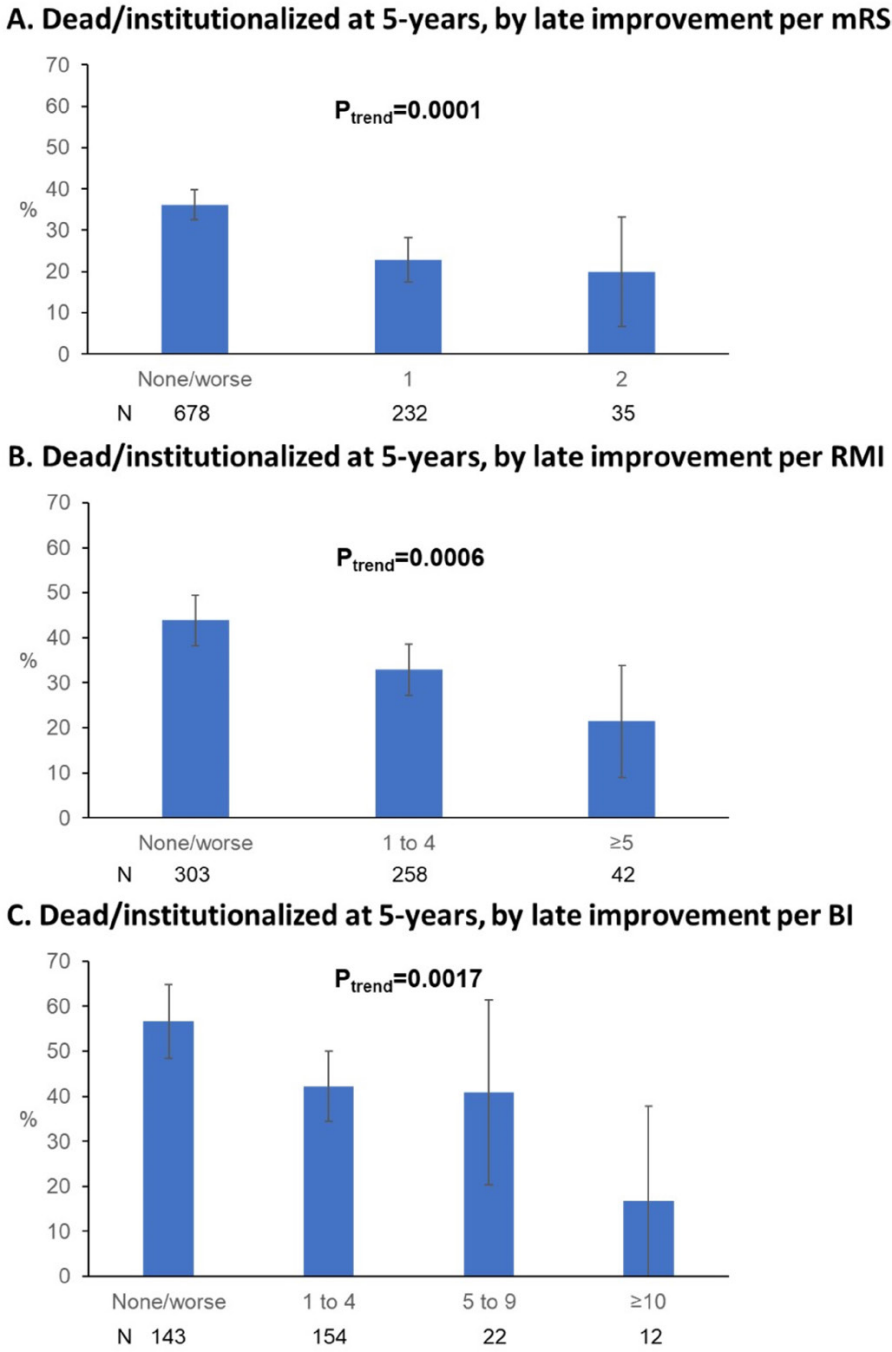
Proportion of 1-year survivors of ischaemic stroke who were dead or newly admitted to a nursing or residential care home by 5 years poststroke, grouped by the degree of improvement in (A) modified Rankin Scale (mRS), (B) Rivermead Mobility Index (RMI) and (C) Barthel Index (BI). Patients who experienced recurrent strokes during follow-up are excluded, as are patients with 3 months mRS=0 (A), RMI=15 (B) and BI=20 (C) who could not demonstrate further improvement. Results of non-parametric tests for trend significance (P_trend_) are shown. Bars represent 95% CIs. N is the number of patients contributing to the denominator in each bar.

Each increment of late improvement per mRS/RMI/BI was also associated with lower 5-year mortality/institutionalisation, even on excluding those with recurrent strokes (figure 3). Late improvement remained associated with lower 5-year mortality/institutionalisation on Cox regressions (eg, aHR with mRS improvement: 0.59, 0.46 to 0.76, p<0.001, table 2), including when excluding recurrent strokes (eg, aHR(mRS): 0.59, 0.44 to 0.79, p<0.001, online supplementary eTable 10) and on further excluding those with premorbid disability and adjusting for other confounders (eg, aHR(mRS): 0.57, 0.41 to 0.79, p=0.001; aHR(RMI/BI): 0.60, 0.43 to 0.84, 0.003, online supplementary eTable 11).

**Table 2:** Cox regression models for the association of functional improvement per the modified Rankin Scale (mRS), Rivermead Mobility Index (RMI) and/or Barthel Index (BI) between 3 months and 1-year poststroke, with 5-year mortality/institutionalisation, adjusted for age, sex, stroke subtype (lacunar vs non-lacunar) and 3-month score on the relevant measure, in 1-year survivors of ischaemic stroke

	Hazard for 5-year mortality/institutionalisation							
	mRS improvement between 3 and 12 months		RMI improvement between 3 and 12 months		BI and/or RMI improvement between 3 and 12 months		mRS, RMI, and/or BI improvement between 3 and 12 months	
	aHR (95% CI)	P>|z|	aHR (95% CI)	P>|z|	aHR (95% CI)	P>|z|	aHR (95% CI)	P>|z|
**Late functional improvement**	0.59 (0.46 to 0.76)	<0.001	0.63 (0.50 to 0.80)	<0.001	0.73 (0.58 to 0.92)	0.008	0.74 (0.61 to 0.91)	0.004
**Age**	1.08 (1.07 to 1.09)	<0.001	1.08 (1.06 to 1.09)	<0.001	1.08 (1.07 to 1.10)	<0.001	1.08 (1.07 to 1.10)	<0.001
**Male sex**	1.12 (0.91 to 1.38)	0.27	1.22 (0.96 to 1.56)	0.11	1.09 (0.86 to 1.38)	0.46	1.11 (0.90 to 1.36)	0.34
**Lacunar stroke**	0.67 (0.50 to 0.90)	0.008	0.62 (0.44 to 0.88)	0.007	0.62 (0.44 to 0.87)	0.006	0.66 (0.49 to 0.89)	0.006
**3 months mRS**	1=Reference		N/A		N/A			
**2**	1.40 (1.01 to 1.94)	0.044					1.76 (0.77 to 4.05)	0.18
**3**	2.96 (2.19 to 4.01)	<0.001					3.82 (1.68 to 8.69)	0.001
**4**	4.12 (2.96 to 5.75)	<0.001					4.98 (2.17 to 11.5)	<0.001
**5**	8.91 (6.07 to 13.1)	<0.001					10.8 (4.6 to 25.3)	<0.001
**3-month RMI**	N/A		0.88 (0.86 to 0.90)	<0.001	N/A		N/A	
**3-month BI**	N/A		N/A		0.89 (0.88 to 0.91)	<0.001	N/A	
	p>|X^2^| n	<0.0001 1135	p>|X^2^| n	<0.0001 706	p>|X^2^| n	<0.0001 747	p>|X^2^| n	<0.0001 1179

These models exclude patients who were already living in a nursing or residential care home pre-stroke and alive at 5 years (n=5) and those who could not show improvement by definition that is, with 3-month mRS=0 (n=135), 3-month RMI=15 (n=369) or 3-month BI=20 (n=661), with 91 patients meeting all three criteria.

N/A, not applicable.

Similarly, patients with late improvement also incurred lower 5-year healthcare/social care costs (margin(mRS): US$17 369, 95% CI −25 271 to −9469, p<0.001, table 3). These associations remained when excluding recurrent strokes (margin(mRS): US$17 283, –25 594 to −8972, p<0.001, online supplementary eTable 12) and accounting for comorbidities, recurrent strokes and excluding those with premorbid mRS >2 (margin(mRS): US$16 439, –24 467 to –8 411, p<0.001; margin(RMI/BI): US$13 490, –23 440 to –3541, p=0.024, online supplementary eTable 13).

**Table 3:** Generalised linear models for the association of functional improvement per the modified Rankin Scale (mRS), Rivermead Mobility Index (RMI) and/or Barthel Index (BI) between 3 months and 1 year poststroke, with 5-year health and social care costs, adjusted for age, sex, stroke subtype (lacunar vs non-lacunar) and 3-month score on the relevant measure, in 1-year survivors of ischaemic stroke

	mRS improvement between 3 and 12 months		RMI improvement between 3 and 12 months		BI and/or RMI improvement between 3 and 12 months		mRS, RMI and/or BI improvement between 3 and 12 months	
	Margin, US$ (95% CI)	P>|z|	Margin, US$ (95% CI)	P>|z|	Margin, US$ (95% CI)	P>|z|	Margin, US$ (95% CI)	P>|z|
**Late functional improvement**	−17 370 (−25 271 to −9469)	<0.001	−11 701 (−20 687 to −2716)	0.011	−9411 (−18 239 to −583)	0.037	−6627 (−12 648 to −606)	0.031
**Age**	796 (505 to 1086)	<0.001	736 (323 to 1149)	<0.001	827 (417 to 1237)	<0.001	801 (526 to 1076)	<0.001
**Male sex**	−4558 (−10 598 to 1481)	0.14	−317 (−8881 to 8247)	0.94	−7153 (−15 702 ot 1296)	0.10	−5295 (−11 111 to 521)	0.074
**Lacunar stroke**	−4972 (−12 331 to 2386)	0.19	−7810 (−18 to 406 to 2786)	0.15	−6372 (−17 196 to 3733)	0.21	−5833 (−12 907 to 1242)	0.11
**3-month mRS**	1=Reference		N/A		N/A			
**2**	4515 (621 to 8409)	0.023					6597 (879 to 12 315)	0.024
**3**	20 257 (12 733 to 27 782)	<0.001					24 080 (15 149 to 33 012)	<0.001
**4**	47 670 (30 062 to 65 331)	<0.001					46 624 (29 752 to 63 497)	<0.001
**5**	85 725 (41 303 to 130 147)	<0.001					81 352 (40 034 to 122 669)	<0.001
**3-month RMI**	N/A		−4925 (−6546 to −3304)	<0.001	N/A		N/A	
**3-month BI**	N/A		N/A		−3658 (−4988 to −2329)	<0.001	N/A	
	n	973	n	606	n	644	n	1006

These models exclude patients without full 5 years of follow-up (n=173), those who were already living in a nursing or residential care home prestroke (n=30), and those who could not show improvement by definition for the respective model that is, with 3-month mRS=0, 3-month RMI=15 or 3-month BI=20.

N/A, not applicable.

When the analyses were limited to patients with 3-month mRS of 2–4, while accounting for recurrent strokes during follow-up, late improvement remained significantly associated with lower 5-year mortality, institutionalisation (aHR for mortality/institutionalisation: 0.67, 0.48 to 0.92, p=0.014) and healthcare/social care costs (margin: US$20 306, –30 211 to -10 402, p<0.001, online supplementary eTable 14).

On additional sensitivity analyses, late improvement per mRS/RMI was associated with lower 5-year mortality/institutionalisation regardless of whether this improvement was observed between 3 and 6 months (aHR(mRS): 0.62, 0.48 to 0.80, p<0.001; aHR(RMI): 0.77, 0.59 to 0.99, p=0.044) or between 6 and 12 months poststroke (aHR(mRS): 0.51, 0.36 to 0.72, p<0.001; aHR(RMI): 0.56, 0.42 to 0.74, p<0.001). Similarly, late improvement per mRS/RMI was associated with lower 5-year costs regardless of whether it occurred between 3 and 6 months (margin(mRS): US$15 759, –23 181 to −8337, p<0.001; margin(RMI): US$12 916, –23 007 to −2825, p=0.012) or 6–12 months (margin(mRS): US$15 221, –24 738 to −5705, p=0.002; margin(RMI): US$14 556, –24 292 to −4821, p=0.003). For the BI, improvement between 6 and 12 months was associated with lower 5-year mortality/institutionalisation (aHR(BI): 0.63, 0.43 to 0.93, p=0.019), whereas improvement between 3–6 months was associated with lower 5-year costs (margin(BI): US$3990, –6887 to −1092, p=0.007).

On coding late improvement between 3 and 12 months as a numerical rather than dichotomous variable, each increment of improvement was associated with lower mortality/institutionalisation (eg, aHR(RMI): 0.85, 0.79 to 0.91, p<0.001, online supplementary eTable 15) and healthcare costs (eg, margin(RMI): US$5603, –8709 to –2498, p<0.001, online supplementary eTable 16).

## Discussion

By prospective assessment of functional status, mortality, institutionalisation and health and social care utilisation in a population-based cohort study, we showed that patients with ischaemic stroke who demonstrate late functional improvement between 3 and 12 months poststroke have better 5-year clinical and health economic outcomes. This association was seen with improvement on any of three different commonly used scales, remained significant in multiple sensitivity analyses, and was not accounted for by differences in 3-month or premorbid disability, comorbidities or recurrent events. Our findings have implications for motivating patients with ischaemic stroke in clinical practice, for cost-effectiveness analyses and coverage of rehabilitative services, and for the development of therapies to promote late recovery.

First, our findings should motivate patients and clinicians to maximise late recovery in routine practice, and consider access to rehabilitative services for at least 1-year poststroke. Physicians should consider this potential for late improvement when discussing prognosis and rehabilitation options. Our results should also incentivise payers to expand coverage for proven late therapies like CIMT^5^ beyond just the first few months poststroke, as such investment may pay off with sustained long-term independence and lower healthcare and social care costs. Given that our study population was relatively elderly, and late improvement was more common among younger patients, the potential for such improvement and the magnitude of associated benefits may be higher in the general patient population.

Second, our findings have implications for health economic analyses of stroke therapies and of the burden of stroke at a population level for resource planning. Cost-effectiveness analyses of acute stroke therapies have assumed a static post-3-month outcome for dependent patients without any potential for improvement.^26^ Accounting for the potential for a state-change from disabled to non-disabled, particularly in the first year poststroke, could add to the robustness of cost-effectiveness models. Furthermore, our results imply that later restorative therapies that promote functional improvement beyond 3 months are likely to be cost-effective, though cost savings will of course be attenuated by the costs of delivering such therapies.

Third, our findings further highlight the importance of understanding and leveraging this phenomenon of late recovery beyond 3 months poststroke. A recent abstract analysed data from three phase-III randomised multicentre trials of acute ischaemic stroke (2555 patients) and found a similar rate of late functional improvement (by ≥1 mRS grades) between 3 and 12 months in about one-fourth of the sample, less often in patients who were older or had more severe strokes.^27^ An analysis of individual patient data from 11 rehabilitation pilot studies demonstrated a gradient of recovery that faded out exponentially and reached asymptotic levels after about 18 months poststroke, further supporting our observation of a longer window for functional improvement extending beyond 3 months.^28^ Growing neuroscientific research implicates multiple, likely time-dependent mechanisms underpinning such recovery, including cortical activation and reorganisation (including contralesionally),^29^ network modulation,^30^ improved interhaemispheric connectivity,^31^ and alteration of axomyelinic synapses to modify myelin properties or recruit companion glia.^32^ Continued research into these mechanisms and therapies to exploit them—like repetitive transcranial magnetic stimulation and transcranial direct current stimulation^19 33^—is essential to optimise outcomes for the over 25% of patients with stroke who remain with significant disability by 3 months poststroke.^8^

Our study has several strengths, including completeness of follow-up (1.3% missing) and generalisability from a population-based sample. The distribution of initial NIHSS scores in our study population is comparable with that reported in other population-based studies. For example, both the Cincinatti/Northern Kentucky Stroke Study and the Brain Attack Surveillance in Corpus Christi Project reported median NIHSS scores of 3 among 2233 and 1796 patients with stroke, respectively, as compared with 2 (mean 3.6) in our study.^34 35^ In addition, we adjusted our analyses of late improvement and 5-year outcomes for 3-month functional status, an important confounder strongly predictive of long-term outcomes, especially given baseline differences between patients with and without late improvement (better 3-month status in the latter in our cohort). Nevertheless, there are some shortcomings. First, a randomised-controlled trial would be required to definitively prove a causal association between promoting late functional improvement and reducing mortality or healthcare costs. Although our findings imply that treatments promoting late improvements will likely improve long-term outcomes, our analyses could not account for the added costs per unit of improvement that would be incurred through interventions beyond standard care. Second, since we did not serially determine neurological impairments using measures like the NIHSS or Fugl-Meyer scale,^36^ we could not differentiate between improvement in impairment and adaptation to impairment, as both can lead to functional improvement. However, since functional improvement is associated with better 5-year outcomes, either process may be worth maximising. Third, scales like the mRS can be confounded by non-stroke-related disability and have inter-rater variability.^12^ However, our findings were similar for the mRS, BI and RMI, and remained significant after adjusting for and/or excluding premorbid disability and comorbidities. Fourth, we could not adjust for all bio-psychosocial factors that might affect improvement and mortality/institutionalisation, such as mood/anxiety, social support, economic status and cognitive impairment. However, many of these factors are reflected in poststroke and premorbid mRS,^37^ which were accounted for in our sensitivity analyses, and we suspect that such interindividual variability is unlikely to have driven the robust associations in our study. Fifth, the majority of our cohort did not receive inpatient or community-based poststroke rehabilitation. Although we do not know the extent to which this was related to differences in impairment, access or patient participation, it may have affected the occurrence of late improvement in our cohort. While not directly relevant to our analysis of late improvement, thrombolysis rates were also relatively low in our cohort, although comparable to rates in the UK during this period.^38^ This may limit the generalisability of our 3-month mRS results to populations with more severe strokes or receiving more aggressive hyperacute treatment.^39^ Our results are also only applicable to patients with ischaemic stroke; similar analyses of late improvement may be quite relevant in survivors of intracerebral haemorrhage.^40^

In conclusion, late functional improvement poststroke is associated with lower 5-year mortality, institutionalisation rates and healthcare/social care costs. These findings should further motivate patients and clinicians to maximise late recovery in routine practice, and consider access to rehabilitative services for at least 1-year poststroke.
