# Supplementary material for: Late functional improvement and 5-year poststroke outcomes: a population-based cohort study
Source: J Neurol Neurosurg Psychiatry. 2020 Jun 23;91(8):831–9. doi: 10.1136/jnnp-2019-322365 (PMC7402458; doi:10.1136/jnnp-2019-322365)
Supplement: Supplementary data [file jnnp-2019-322365supp001.pdf]

ONLINE SUPPLEMENT

Supplemental Tables

eTable 1. Characteristics of 1-year survivors of ischaemic stroke who did and did not demonstrate functional improvement per the Rivermead Mobility Index (RMI) between 3-months and 1-year post-stroke. Only patients with 3-month RMI<15 (capable of showing further improvement) are included.

**Characteristic**	**Late improvement** (n=351)	**No late improvement** (n=356)	**P-value**
**Age, mean (standard deviation)**	73.3 (12.3)	77.5 (9.7)	<0.0001*
**Sex–male(%)**	175 (49.9)	150 (42.1)	0.039*
**Previous history(%):**			
Myocardial Infarction	46 (13.1)	50 (14.0)	0.72
Angina	55 (15.7)	69 (19.4)	0.19
Atrial Fibrillation	78 (22.2)	66 (18.5)	0.22
Hypertension	234 (66.7)	254 (71.4)	0.18
Dyslipidemia	126 (35.9)	131 (36.8)	0.80
Diabetes	64 (18.2)	54 (15.2)	0.27
Peripheral Vascular Disease	26 (7.4)	26 (7.3)	0.96
Stroke	44 (12.5)	41 (11.5)	0.68
Transient Ischaemic Attack	46 (13.1)	64 (18.0)	0.07
Smoking	200 (57.0)	205 (57.6)	0.87
Heart Failure	28 (8.0)	38 (10.7)	0.22
Valvular Heart Disease	46 (13.1)	37 (10.4)	0.26
Cancer	55 (15.7)	50 (14.0)	0.54
Pre-stroke mRS>2	53 (15.1)	69 (19.4)	0.13
Pre-stroke BI<20	72 (20.7)	102 (29.1)	0.010*
**Initial NIHSS**, mean (SD)	4.1 (4.5)	4.2 (5.4)	0.35
**TOAST subtype (%)**			
Lacunar (small vessel disease)	78 (22.2)	52 (14.6)	0.009*
Cardio-embolism	87 (27.0)	83 (26.2)	0.81
Large artery atherosclerosis	41 (12.7)	38 (12.0)	0.78
Undetermined	68 (21.1)	93 (29.3)	0.017*
Unknown	16 (5.0)	41 (12.9)	<0.001*
**Received thrombolysis (%)**	6 (1.9)	5 (1.6)	0.77
**Received in-hospital rehabilitation (%)**	133 (37.9)	120 (33.7)	0.25
Length of stay for rehabilitation (median days, IQR)	33 (16-68)	42.5 (16.5-118)	0.036*
Timing of in-hospital rehab completion, median days post-stroke (IQR)	31 (9-71)	33 (9-109)	0.14
**Received community-based rehabilitation (%)**	59 (16.8)	57 (16.0)	0.78
Number of sessions, median (IQR)	3 (1-9)	2 (1-4)	0.14
Timing of rehabilitation completion, median days post-stroke (IQR)	551 (229-1288)	925 (380-1307)	0.11
**Received hospital- or community-based rehab between 3-12 months (%)**	40 (11.4)	43 (12.1)	0.78
**3-month RMI,** median (IQR)	11 (7-13)	12 (8-14)	0.0055*
**Late RMI improvement timing (%)†**			
3-6 months post-stroke	243 (69.2)	0	<0.0001*
6-12 months post-stroke	157 (44.7)	0	<0.0001*
**Recurrent stroke** within 5-years (%)	51 (14.5)	67 (18.8)	0.13
**Any recurrent vascular event** within 5-years(%)	94 (26.8)	111 (31.2)	0.20

**Post-stroke depression**(%)	111 (31.6)	132 (37.1)	0.13

†49 patients showed improvement on the RMI both between 3 to 6 months and further between 6 months and 12 months.
Significant differences ($p<0.05$) are indicated by an asterisk(*). We compared ordinal/continuous variables using the Wilcoxon rank-sum (Mann-Whitney U) and dichotomous variables using chi-squared tests. Abbreviations: mRS – modified Rankin scale, NIHSS – National Institutes of Health Stroke Scale, RMI – Rivermead Mobility Index, TOAST – Trial of Org10172 in Acute Stroke Treatment, IQR – inter-quartile range.

eTable 2. Characteristics of 1-year survivors of ischaemic stroke who did and did not demonstrate functional improvement per the Barthel Index (BI) between 3-months and 1-year post-stroke. Only patients with 3-month BI<20 are included.

Characteristic	Late improvement (n=158)	No late improvement (n=293)	P-value
**Age, mean (standard deviation)**	76.4 (10.4)	77.3 (10.5)	0.25
**Sex–male(%)**	76 (48.1)	123 (42.0)	0.21
**Previous history(%):**			
Myocardial Infarction	21 (13.3)	46 (15.7)	0.49
Angina	22 (13.9)	57 (19.5)	0.14
Atrial Fibrillation	39 (24.7)	59 (20.1)	0.26
Hypertension	113 (71.5)	207 (70.7)	0.84
Dyslipidemia	44 (27.9)	113 (38.6)	0.023*
Diabetes	24 (15.2)	59 (20.1)	0.20
Peripheral Vascular Disease	17 (10.8)	16 (5.5)	0.039*
Stroke	16 (10.1)	49 (16.7)	0.057
Transient Ischaemic Attack	19 (12.0)	55 (18.8)	0.065
Smoking	88 (55.7)	151 (51.5)	0.40
Heart Failure	11 (6.7)	36 (12.30	0.077
Valvular Heart Disease	14 (8.9)	42 (14.3)	0.093
Cancer	32 (20.3)	52 (17.8)	0.51
Pre-stroke mRS>2	32 (20.3)	83 (28.3)	0.061
Pre-stroke BI<20	43 (27.7)	113 (39.4)	0.015*
**Initial NIHSS**, mean (SD)	6.7 (6.3)	4.5 (5.3)	<0.0001*
**TOAST subtype (%)**			
Lacunar (small vessel disease)	24 (15.2)	51 (17.4)	0.55
Cardio-embolism	44 (30.8)	71 (27.2)	0.45
Large artery atherosclerosis	17 (11.9)	27 (10.3)	0.63
Undetermined	29 (20.3)	67 (25.7)	0.22
Unknown	16 (11.2)	35 (13.4)	0.52
**Received thrombolysis (%)**	4 (2.7)	6 (2.3)	0.78
**Received in-hospital rehabilitation (%)**	101 (63.9)	118 (40.3)	<0.001*
Length of stay for rehabilitation, median days (IQR)	58 (28-98)	42 (17-87)	0.091
Timing of in-hospital rehab completion, median days post-stroke (IQR)	60 (22-105)	34 (13-97)	0.041*
**Received community-based rehabilitation (%)**	32 (20.3)	46 (15.7)	0.22
Number of sessions, median (IQR)	3 (1-10.5)	2.5 (1-6)	0.36
Timing of rehabilitation completion, median days post-stroke (IQR)	597 (236-1273)	925 (316-1362)	0.40
**Received hospital- or community-based rehab between 3-12 months (%)**	35 (22.2)	34 (11.6)	0.003*
**3-month BI,** median (IQR)	13 (8-17)	18 (13-19)	<0.0001*
**Late BI improvement timing (%)**†			
3-6 months post-stroke	121 (76.6)	0	<0.0001*
6-12 months post-stroke	43 (27.2)	0	<0.0001*
**Recurrent stroke** within 5-years (%)	16 (10.1)	54 (18.4)	0.020*
**Any recurrent vascular event** within 5-years(%)	30 (19.0)	99 (33.8)	0.001*
**Post-stroke depression**(%)	67 (42.4)	109 (37.2)	0.28

†6 patients showed improvement on the BI both between 3 and 6 months and between 6 months and 1 year post-stroke.
Significant differences ($p<0.05$) are indicated by an asterisk(*). We compared ordinal/continuous variables using the Wilcoxon rank-sum (Mann-Whitney U) and dichotomous variables using chi-squared tests. Abbreviations: mRS – modified Rankin scale, BI – Barthel Index, NIHSS – National Institutes of Health Stroke Scale, TOAST – Trial of Org10172 in Acute Stroke Treatment, IQR – inter-quartile range.

eTable 3. Cox regression models for the association of functional improvement per the modified Rankin Scale (mRS), Rivermead Mobility Index (RMI), and/or Barthel Index (BI) between 3-months to 1-year post-stroke, with 5-year mortality, adjusted for age, sex, stroke subtype (lacunar vs non-lacunar), and 3-month score on the relevant measure, in 1-year survivors of ischaemic stroke. These models exclude patients who could not show improvement by definition, namely those with 3-month mRS=0 (n=135), 3-month RMI=15 (n=369), or 3-month BI=20 (n=661), with 91 patients meeting all three criteria.

	**Hazard for 5-year mortality**							
	**mRS improvement between 3-12 months**		**RMI improvement between 3-12 months**		**BI and/or RMI improvement between 3-12 months**		**mRS, RMI, and/or BI improvement between 3-12 months**	
	**aHR(95%CI)**	**p>|z|**	**aHR(95%CI)**	**p>|z|**	**aHR(95%CI)**	**p>|z|**	**aHR(95%CI)**	**p>|z|**
**Late functional improvement**	0.68 (0.51-0.91)	0.009	0.64 (0.49-0.85)	0.002	0.70 (0.54-0.91)	0.009	0.66 (0.52-0.83)	<0.001
**Age**	1.08 (1.07-1.10)	<0.001	1.08 (1.07-1.10)	<0.001	1.09 (1.07-1.10)	<0.001	1.09 (1.07-1.10)	<0.001
**Male sex**	1.21 (0.96-1.54)	0.11	1.39 (1.05-1.85)	0.022	1.27 (0.97-1.66)	0.082	1.21 (0.96-1.53)	0.11
**Lacunar stroke**	0.77 (0.55-1.08)	0.12	0.67 (0.45-1.01)	0.058	0.71 (0.47-1.05)	0.087	0.76 (0.55-1.06)	0.11
**3-month mRS**	1=Reference		N/A		N/A			
**2**	1.53 (1.07-2.21)	0.021					1.48 (0.54-3.41)	0.36
**3**	2.55 (1.82-3.59)	<0.001					2.53 (1.10-5.80)	0.028
**4**	2.68 (1.82-3.93)	<0.001					2.46 (1.05-5.75)	0.037
**5**	3.62 (2.28-5.74)	<0.001					3.41 (1.41-8.25)	0.007
**3-month RMI**	N/A		0.93 (0.90-0.96)	<0.001	N/A		N/A	
**3-month BI**	N/A		N/A		0.95 (0.93-0.97)	<0.001	N/A	
	$p>\|X^2\|$	<0.0001	$p>\|X^2\|$	<0.0001	$p>\|X^2\|$	<0.0001	$p>\|X^2\|$	<0.0001
	n	1,135	n	706	n	747	n	1,179

eTable 4. Cox regression models for the association of functional improvement per the modified Rankin Scale (mRS), Rivermead Mobility Index (RMI), and/or Barthel Index (BI) between 3-months to 1-year post-stroke, with 5-year mortality, adjusted for age, sex, stroke subtype (lacunar vs non-lacunar), and 3-month score on the relevant measure, in 1-year survivors of ischaemic stroke, excluding those with recurrent strokes during follow-up. These models also exclude patients who could not show improvement by definition, namely those with 3-month mRS=0 (n=135), 3-month RMI=15 (n=369), or 3-month BI=20 (n=661), with 91 patients meeting all three criteria.

	Hazard for 5-year mortality							
	mRS improvement between 3-12 months		RMI improvement between 3-12 months		BI and/or RMI improvement between 3-12 months		mRS, RMI, and/or BI improvement between 3-12 months	
	aHR(95%CI)	p>|z|	aHR(95%CI)	p>|z|	aHR(95%CI)	p>|z|	aHR(95%CI)	p>|z|
**Late functional improvement**	0.69 (0.49-0.96)	0.026	0.64 (0.46-0.87)	0.004	0.68 (0.51-0.91)	0.010	0.65 (0.50-0.85)	0.001
**Age**	1.09 (1.07-1.10)	<0.001	1.09 (1.07-1.12)	<0.001	1.09 (1.07-1.12)	<0.001	1.09 (1.07-1.11)	<0.001
**Male sex**	1.24 (0.95-1.62)	0.11	1.51 (1.09-2.08)	0.013	1.41 (1.04-1.91)	0.027	1.26 (0.97-1.63)	0.088
**Lacunar stroke**	0.86 (0.59-1.23)	0.40	0.77 (0.50-1.20)	0.25	0.81 (0.53-1.24)	0.34	0.86 (0.60-1.23)	0.40
**3-month mRS**			N/A		N/A			
**2**	1.40 (0.94-2.09)	0.097					1.88 (0.68-5.22)	0.23
**3**	2.33 (1.61-3.36)	<0.001					3.21 (1.17-8.81)	0.024
**4**	2.29 (1.49-3.50)	<0.001					2.93 (1.04-8.23)	0.041
**5**	2.98 (1.78-4.99)	<0.001					3.89 (1.33-11.4)	0.013
**3-month RMI**	N/A		0.92 (0.90-0.97)	<0.001			N/A	
**3-month BI**	N/A				0.95 (0.93-0.98)	<0.001	N/A	
	$p>\|X^2\|$	<0.0001	$p>\|X^2\|$	<0.0001	$p>\|X^2\|$	<0.0001	$p>\|X^2\|$	0.001
	n	965	n	588	n	625	n	1,003

eTable 5. Cox regression models for the association of functional improvement per the modified Rankin Scale (mRS), Rivermead Mobility Index (RMI), and/or Barthel Index (BI) between 3-months to 1-year post-stroke, with 5-year mortality, adjusted for age, sex, stroke subtype (lacunar vs non-lacunar), 3-month score on the relevant measure, and recurrent strokes during follow-up in 1-year survivors of ischaemic stroke, excluding those with pre-stroke disability (mRS>2, and also BI<20 for analyses involving RMI/BI). Each model is also further adjusted for any additional characteristics that differed significantly between patients who did or did not demonstrate late improvement per that parameter (these hazards are not shown): for mRS, this included prior TIAs and angina; for BI, this included dyslipidemia, peripheral vascular disease, and inpatient rehabilitation. These models also exclude patients who could not show improvement by definition, namely those with 3-month mRS=0 (n=135), 3-month RMI=15 (n=369), or 3-month BI=20 (n=661), with 91 patients meeting all three criteria.

	**Hazard for 5-year mortality**							
	**mRS improvement between 3-12 months**		**RMI improvement between 3-12 months**		**BI and/or RMI improvement between 3-12 months**		**mRS, RMI, and/or BI improvement between 3-12 months**	
	**aHR(95%CI)**	**p>|z|**	**aHR(95%CI)**	**p>|z|**	**aHR(95%CI)**	**p>|z|**	**aHR(95%CI)**	**p>|z|**
**Late functional improvement**	0.71 (0.51-0.99)	0.040	0.67 (0.47-0.97)	0.032	0.80 (0.58-1.12)	0.19	0.68 (0.51-0.90)	0.006
**Age**	1.09 (1.07-1.10)	<0.001	1.09 (1.06-1.11)	<0.001	1.08 (1.06-1.10)	<0.001	1.09 (1.07-1.11)	<0.001
**Male sex**	1.35 (1.01-1.79)	0.040	1.52 (1.06-2.20)	0.024	1.46 (1.04-2.05)	0.030	1.35 (1.02-1.79)	0.033
**Lacunar stroke**	0.79 (0.54-1.16)	0.23	0.65 (0.38-1.09)	0.103	0.65 (0.40-1.07)	0.090	0.80 (0.55-1.16)	0.24
**3-month mRS**			N/A		N/A			
**2**	1.56 (1.08-2.26)	0.019					1.51 (0.65-3.51)	0.34
**3**	2.43 (1.65-3.60)	<0.001					2.43 (1.03-5.72)	0.042
**4**	1.79 (1.07-3.01)	0.027					1.74 (0.70-4.34)	0.24
**5**	2.97 (1.70-5.20)	<0.001					2.86 (1.12-7.32)	0.028
**3-month RMI**	N/A		0.93 (0.89-0.96)	<0.001	N/A		N/A	
**3-month BI**	N/A		N/A		0.97 (0.94-0.99)	0.041	N/A	
**Recurrent strokes**	1.28 (0.92-1.80)	0.15	1.12 (0.72-1.73)	0.61	1.18 (0.78-1.79)	0.43	1.28 (0.92-1.79)	0.14
	$p>\|X^2\|$	<0.0001	$p>\|X^2\|$	<0.0001	$p>\|X^2\|$	<0.0001	$p>\|X^2\|$	<0.0001
	n	963	n	501	n	559	n	1,007

eTable 6. Cox regression models for the association of functional improvement per the modified Rankin Scale (mRS), Rivermead Mobility Index (RMI), and/or Barthel Index (BI) between 3-months to 1-year post-stroke, with 1-year institutionalization, adjusted for age, sex, stroke subtype (lacunar vs non-lacunar), and 3-month score on the relevant measure, in 1-year survivors of ischaemic stroke. These models exclude patients who were already living in a nursing or residential care home pre-stroke (n=30) and those who could not show improvement by definition, namely those with 3-month mRS=0 (n=135), 3-month RMI=15 (n=369), or 3-month BI=20 (n=661), with 91 patients meeting all three criteria.

	**Hazard for 1-year institutionalization**							
	**mRS improvement between 3-12 months**		**RMI improvement between 3-12 months**		**BI and/or RMI improvement between 3-12 months**		**mRS, RMI, and/or BI improvement between 3-12 months**	
	**aHR(95%CI)**	**p>|z|**	**aHR(95%CI)**	**p>|z|**	**aHR(95%CI)**	**p>|z|**	**aHR(95%CI)**	**p>|z|**
**Late functional improvement**	0.32 (0.16-0.63)	0.001	0.41 (0.24-0.71)	0.001	0.63 (0.39-1.02)	0.060	0.57 (0.37-0.90)	0.014
**Age**	1.08 (1.05-1.11)	<0.001	1.06 (1.03-1.10)	<0.001	1.08 (1.04-1.11)	<0.001	1.09 (1.05-1.12)	<0.001
**Male sex**	1.03 (0.63-1.67)	0.91	1.14 (0.64-2/04)	0.66	0.92 (0.54-1/58)	0.77	1.07 (0.66-1.74)	0.79
**Lacunar stroke**	0.24 (0.09-0.66)	0.006	0.26 (0.08-0.83)	0.023	0.28 (0.09-0.88)	0.030	0.23 (0.09-0.64)	0.005
**3-month mRS**	1=Reference		N/A		N/A			
**2**	1.28 (0.26-6.34)	0.77					2.80 (0.29-26.9)	0.37
**3**	7.86 (2.29-26.9)	0.001					15.8 (2.11-119)	0.007
**4**	32.5 (9.9-107)	<0.001					58.6 (8.01-429)	<0.001
**5**	104 (31.5-344)	<0.001					200 (27.2-1480)	<0.0011
**3-month RMI**	N/A		0.72 (0.68-0.77)	<0.001	N/A		N/A	
**3-month BI**	N/A		N/A		0.81 (0.78-0.84)	<0.001	N/A	
	p>|X^2|	<0.0001	p>|X^2|	<0.0001	p>|X^2|	<0.0001	p>|X^2|	<0.0001
	n	1,109	n	692	n	732	n	1,153

eTable 7. Cox regression models for the association of functional improvement per the modified Rankin Scale (mRS), Rivermead Mobility Index (RMI), and/or Barthel Index (BI) between 3-months to 1-year post-stroke, with 5-year institutionalization, adjusted for age, sex, stroke subtype (lacunar vs non-lacunar), and 3-month score on the relevant measure, in 1-year survivors of ischaemic stroke. These models exclude patients who were already living in a nursing or residential care home pre-stroke (n=30) and those who could not show improvement by definition i.e. with 3-month mRS=0 (n=135), 3-month RMI=15 (n=369), or 3-month BI=20 (n=661), with 91 patients meeting all three criteria.

	**Hazard for 5-year institutionalization**							
	**mRS improvement between 3-12 months**		**RMI improvement between 3-12 months**		**BI and/or RMI improvement between 3-12 months**		**mRS, RMI, and/or BI improvement between 3-12 months**	
	**aHR(95%CI)**	**p>|z|**	**aHR(95%CI)**	**p>|z|**	**aHR(95%CI)**	**p>|z|**	**aHR(95%CI)**	**p>|z|**
**Late functional improvement**	0.48 (0.33-0.72)	<0.001	0.48 (0.34-0.68)	<0.001	0.67 (0.48-0.93)	0.016	0.70 (0.52-0.94)	0.017
**Age**	1.08 (1.06-1.10)	<0.001	1.07 (1.04-1.09)	<0.001	1.08 (1.05-1.10)	<0.001	1.09 (1.07-1.11)	<0.001
**Male sex**	0.98 (0.71-1.36)	0.92	1.05 (0.73-1.52)	0.80	0.88 (0.62-1.26)	0.49	0.96 (0.70-1.31)	0.78
**Lacunar stroke**	0.59 (0.38-0.93)	0.022	0.64 (0.39-1.05)	0.076	0.62 (0.38-1.02)	0.059	0.57 (0.37-0.90)	0.016
**3-month mRS**	1=Reference		N/A		N/A			
**2**	1.12 (0.61-2.05)	0.71					2.73 (0.37-20.4)	0.33
**3**	3.79 (2.31-6.23)	<0.001					9.49 (1.31-68.9)	0.026
**4**	8.13 (4.90-13.5)	<0.001					18.8 (2.49-136)	0.004
**5**	21.7 (12.7-37.3)	<0.001					51.2 (6.98-375)	<0.001
**3-month RMI**	N/A		0.82 (0.80-0.85)	<0.001	N/A		N/A	
**3-month BI**	N/A		N/A		0.86 (0.84-0.88)	<0.001	N/A	
	p>|X^2|	<0.0001	p>|X^2|	<0.0001	p>|X^2|	<0.0001	p>|X^2|	<0.0001
	n	1,109	n	692	n	732	n	1,153

eTable 8. Cox regression models for the association of functional improvement per the modified Rankin Scale (mRS), Rivermead Mobility Index (RMI), and/or Barthel Index (BI) between 3-months to 1-year post-stroke, with 5-year institutionalization, adjusted for age, sex, stroke subtype (lacunar vs non-lacunar), and 3-month score on the relevant measure, in 1-year survivors of ischaemic stroke, excluding those with recurrent strokes during follow-up. These models exclude patients who were already living in a nursing or residential care home pre-stroke (n=30) and those who could not show improvement by definition i.e. with 3-month mRS=0 (n=135), 3-month RMI=15 (n=369), or 3-month BI=20 (n=661), with 91 patients meeting all three criteria.

	**Hazard for 5-year institutionalization**							
	**mRS improvement between 3-12 months**		**RMI improvement between 3-12 months**		**BI and/or RMI improvement between 3-12 months**		**mRS, RMI, and/or BI improvement between 3-12 months**	
	**aHR(95%CI)**	**p>|z|**	**aHR(95%CI)**	**p>|z|**	**aHR(95%CI)**	**p>|z|**	**aHR(95%CI)**	**p>|z|**
**Late functional improvement**	0.46 (0.29-0.73)	0.001	0.48 (0.32-0.71)	<0.001	0.63 (0.44-0.92)	0.016	0.68 (0.48-0.96)	0.029
**Age**	1.08 (1.06-1.10)	<0.001	1.07 (1.04-1.09)	<0.001	1.08 (1.05-1.11)	<0.001	1.08 (1.06-1.11)	<0.001
**Male sex**	1.08 (1.06-1.10)	<0.001	1.09 (0.72-1.66)	0.69	0.97 (0.65-1.45)	0.87	1.02 (0.70-1.48)	0.91
**Lacunar stroke**	0.47 (0.27-0.81)	0.007	0.48 (0.26-0.88)	0.018	0.48 (0.26-0.88)	0.018	0.45 (0.25-0.80)	0.006
**3-month mRS**	1=Reference		N/A		N/A			
**2**	1.25 (0.59-2.64)	0.56					0.97 (0.43-2.20)	0.94
**3**	4.91 (2.70-8.92)	<0.001					3.96 (2.07-7.55)	<0.001
**4**	10.3 (5.60-18.8)	<0.001					7.70 (4.03-14.7)	<0.001
**5**	25.5 (13.4-48.3)	<0.001					19.2 (9.67-38.0)	<0.001
**3-month RMI**	N/A		0.82 (0.79-0.85)	<0.001	N/A		N/A	
**3-month BI**	N/A		N/A		0.85 (0.83-0.88)	<0.001	N/A	
	$p>\|X^2\|$	<0.0001	$p>\|X^2\|$	<0.0001	$p>\|X^2\|$	<0.0001	$p>\|X^2\|$	<0.0001
	n	945	n	580	n	616	n	711

eTable 9. Cox regression models for the association of functional improvement per the modified Rankin Scale (mRS), Rivermead Mobility Index (RMI), and/or Barthel Index (BI) between 3-months to 1-year post-stroke, with 5-year institutionalization, adjusted for age, sex, stroke subtype (lacunar vs non-lacunar), and 3-month score on the relevant measure, in 1-year survivors of ischaemic stroke, excluding those with pre-stroke disability (mRS>2, and also BI<20 for analyses involving RMI/BI) or recurrent strokes during follow-up. Each model is also further adjusted for any additional characteristics that differed significantly between patients who did or did not demonstrate late improvement per that parameter (these hazards are not shown): for mRS, this included prior TIAs and angina; for BI, this included dyslipidemia, peripheral vascular disease, initial NIHSS score, and inpatient rehabilitation. These models also exclude patients who were already living in a nursing or residential care home pre-stroke (n=30) and those who could not show improvement by definition i.e. with 3-month mRS=0 (n=135), 3-month RMI=15 (n=369), or 3-month BI=20 (n=661), with 91 patients meeting all three criteria.

	**Hazard for 5-year institutionalization**							
	**mRS improvement between 3-12 months**		**RMI improvement between 3-12 months**		**BI and/or RMI improvement between 3-12 months**		**mRS, RMI, and/or BI improvement between 3-12 months**	
	**aHR(95%CI)**	**p>|z|**	**aHR(95%CI)**	**p>|z|**	**aHR(95%CI)**	**p>|z|**	**aHR(95%CI)**	**p>|z|**
**Late functional improvement**	0.39 (0.23-0.65)	<0.001	0.48 (0.29-0.79)	0.004	0.48 (0.30-0.78)	0.003	0.56 (0.37-0.85)	0.006
**Age**	1.09 (1.06-1.12)	<0.001	1.08 (1.04-1.12)	<0.001	1.08 (1.05-1.12)	<0.001	1.09 (1.06-1.12)	<0.001
**Male sex**	0.94 (0.60-1.46)	0.78	1.07 (0.63-1.81)	0.80	0.99 (0.59-1.68)	0.97	0.94 (0.60-1.48)	0.79
**Lacunar stroke**	0.40 (0.21-0.76)	0.005	0.39 (0.17-0.92)	0.032	0.39 (0.17-0.91)	0.030	0.38 (0.19-0.75)	0.005
**3-month mRS**	1=Reference		N/A		N/A			
**2**	1.29 (0.60-2.78)	0.51					1.05 (0.45-2.43)	0.92
**3**	5.24 (2.74-10.0)	<0.001					4.37 (2.16-8.87)	<0.001
**4**	12.9 (6.67-24.8)	<0.001					10.0 (4.95-20.2)	<0.001
**5**	28.0 (14.3-54.8)	<0.001					22.0 (10.8-44.9)	<0.001
**3-month RMI**	N/A		0.79 (0.75-0.83)	<0.001	N/A		N/A	
**3-month BI**	N/A		N/A		0.85 (0.81-0.89)	<0.001	N/A	
	$p>\|X^2\|$	<0.0001	$p>\|X^2\|$	<0.0001	$p>\|X^2\|$	<0.0001	$p>\|X^2\|$	<0.0001
	n	818	n	416	n	465	n	585

eTable 10. Cox regression models for the association of functional improvement per the modified Rankin Scale (mRS), Rivermead Mobility Index (RMI), and/or Barthel Index (BI) between 3-months to 1-year post-stroke, with 5-year mortality/institutionalization, adjusted for age, sex, stroke subtype (lacunar vs non-lacunar), and 3-month score on the relevant measure, in 1-year survivors of ischaemic stroke, excluding those with recurrent strokes during follow-up. These models exclude patients who were already living in a nursing or residential care home pre-stroke and alive at 5-years (n=5) and those who could not show improvement by definition i.e. with 3-month mRS=0 (n=135), 3-month RMI=15 (n=369), or 3-month BI=20 (n=661), with 91 patients meeting all three criteria.

	**Hazard for 5-year mortality/institutionalization**							
	**mRS improvement between 3-12 months**		**RMI improvement between 3-12 months**		**BI and/or RMI improvement between 3-12 months**		**mRS, RMI, and/or BI improvement between 3-12 months**	
	**aHR(95%CI)**	**p>|z|**	**aHR(95%CI)**	**p>|z|**	**aHR(95%CI)**	**p>|z|**	**aHR(95%CI)**	**p>|z|**
**Late functional improvement**	0.59 (0.44-0.79)	<0.001	0.64 (0.49-0.84)	0.001	0.72 (0.56-0.94)	0.014	0.75 (0.60-0.94)	0.014
**Age**	1.08 (1.07-1.10)	<0.001	1.08 (1.07-1.10)	<0.001	1.09 (1.07-1.11)	<0.001	1.09 (1.07-1.10)	<0.001
**Male sex**	1.16 (0.92-1.46)	0.22	1.28 (0.97-1.68)	0.081	1.21 (0.93-1.57)	0.16	1.15 (0.91-1.45)	0.24
**Lacunar stroke**	0.63 (0.46-0.88)	0.007	0.57 (0.39-0.84)	0.005	0.58 (0.40-0.85)	0.005	0.63 (0.46-0.87)	0.006
**3-month mRS**	1=Reference		N/A		N/A			
**2**	1.36 (0.94-1.96)	0.10					2.19 (0.79-6.05)	0.13
**3**	3.01 (2.16-4.18)	<0.001					4.94 (1.81-13.5)	0.002
**4**	3.83 (2.65-5.53)	<0.001					5.96 (2.16-16.4)	0.001
**5**	8.12 (5.32-12.4)	<0.001					12.5 (4.44-35.3)	<0.001
**3-month RMI**	N/A		0.89 (0.85-0.90)	<0.001	N/A		N/A	
**3-month BI**	N/A		N/A		0.89 (0.88-0.91)	<0.001	N/A	
	$p>\|X^2\|$	<0.0001	$p>\|X^2\|$	<0.0001	$p>\|X^2\|$	<0.0001	$p>\|X^2\|$	<0.0001
	n	965	n	588	n	625	n	1,003

eTable 11. Cox regression models for the association of functional improvement per the modified Rankin Scale (mRS), Rivermead Mobility Index (RMI), and/or Barthel Index (BI) between 3-months to 1-year post-stroke, with 5-year mortality/institutionalization, adjusted for age, sex, stroke subtype (lacunar vs non-lacunar), and 3-month score on the relevant measure in 1-year survivors of ischaemic stroke, excluding those with pre-stroke disability (mRS>2, and also BI<20 for analyses involving RMI/BI) or recurrent strokes during follow-up. Each model is also further adjusted for any additional characteristics that differed significantly between patients who did or did not demonstrate late improvement per that parameter (these hazards are not shown): for mRS, this included prior TIAs and angina; for BI, this included dyslipidemia, peripheral vascular disease, initial NIHSS, and inpatient rehabilitation. These models also exclude patients who were already living in a nursing or residential care home pre-stroke and alive at 5-years (n=5) and those who could not show improvement by definition i.e. with 3-month mRS=0 (n=135), 3-month RMI=15 (n=369), or 3-month BI=20 (n=661), with 91 patients meeting all three criteria.

	**Hazard for 5-year mortality/institutionalization**							
	**mRS improvement between 3-12 months**		**RMI improvement between 3-12 months**		**BI and/or RMI improvement between 3-12 months**		**mRS, RMI, and/or BI improvement between 3-12 months**	
	**aHR(95%CI)**	**p>|z|**	**aHR(95%CI)**	**p>|z|**	**aHR(95%CI)**	**p>|z|**	**aHR(95%CI)**	**p>|z|**
**Late functional improvement**	0.57 (0.41-0.79)	0.001	0.62 (0.44-0.88)	0.008	0.60 (0.43-0.84)	0.003	0.72 (0.55-0.94)	0.016
**Age**	1.09 (1.07-1.11)	<0.001	1.09 (1.07-1.12)	<0.001	1.10 (1.07-1.12)	<0.001	1.10 (1.08-1.12)	<0.001
**Male sex**	1.19 (0.90-1.57)	0.23	1.42 (0.99-2.03)	0.053	1.28 (0.90-1.80)	0.17	1.17 (0.89-1.55)	0.27
**Lacunar stroke**	0.61 (0.42-0.88)	0.009	0.49 (0.29-0.82)	0.007	0.49 (0.30-0.80)	0.004	0.62 (0.43-0.89)	0.009
**3-month mRS**			N/A		N/A			
**2**	1.44 (0.99-2.08)	0.057					2.39 (0.86-6.61)	0.094
**3**	3.00 (2.05-4.38)	<0.001					5.02 (1.80-14.0)	0.002
**4**	3.90 (2.48-6.15)	<0.001					6.32 (2.21-18.1)	0.001
**5**	8.53 (5.23-13.9)	<0.001					13.4 (4.61-38.9)	<0.001
**3-month RMI**	N/A		0.62 (0.44-0.88)	0.008	N/A		N/A	
**3-month BI**	N/A		N/A		0.89 (0.86-0.92)	<0.001	N/A	
	$p>\|X^2\|$	<0.0001	$p>\|X^2\|$	<0.0001	$p>\|X^2\|$	<0.0001	$p>\|X^2\|$	<0.0001
	n	820	n	418	n	465	n	858

eTable 12. Generalized linear models for the association of functional improvement per the modified Rankin Scale (mRS), Rivermead Mobility Index (RMI), and/or Barthel Index (BI) between 3-months to 1-year post-stroke, with 5-year health and social care costs, adjusted for age, sex, stroke subtype (lacunar vs non-lacunar), and 3-month score on the relevant measure, in 1-year survivors of ischaemic stroke, excluding those with recurrent strokes during follow-up. These models also exclude patients without full five years of follow-up (n=173), those who were already living in a nursing or residential care home pre-stroke (n=30), and those who could not show improvement by definition for the respective model i.e. with 3-month mRS=0, 3-month RMI=15, or 3-month BI=20.

	**mRS improvement between 3-12 months**		**RMI improvement between 3-12 months**		**BI and/or RMI improvement between 3-12 months**		**mRS, RMI, and/or BI improvement between 3-12 months**	
	**Margin, $ (95%CI)**	**p>|z|**	**Margin, $ (95%CI)**	**p>|z|**	**Margin, $ (95%CI)**	**p>|z|**	**Margin, $ (95%CI)**	**p>|z|**
**Late functional improvement**	-17,283 (-25,594 to -8,972)	<0.001	-14,331 (-24,635 to -4,027)	0.006	-10,332 (-20,041 to -623)	0.037	-7,590 (-13,998 to -1,181)	0.020
**Age**	626 (344-909)	<0.001	577 (143-1,010)	0.009	646 (225-1,068)	0.003	628 (364-891)	<0.001
**Male sex**	-1,477 (-7,587-4,632)	0.64	3,067 (-6,394-12,528)	0.53	-2,622 (-11,655-6,410)	0.57	-2,079 (-7,890-3,731)	0.48
**Lacunar stroke**	-8,071 (-15,792 to -351)	0.040	-14,312 (-26,207 to -2,417)	0.018	-12,149 (-23,629 to -688)	0.038	-9,167 (-16,500 to -1,833)	0.014
**3-month mRS**	1=Reference		N/A		N/A			
**2**	3,812 (57-7,566)	0.047					7,290 (2,677-11,903)	0.002
**3**	21,173 (12,895-29,450)	<0.001					26,955 (17,721-36,188)	<0.001
**4**	46,132 (27,124-65,141)	<0.001					47,555 (29,502-65,608)	<0.001
**5**	79,244 (32,719-125,769)	0.001					78,052 (34,741-121,362)	<0.001
**3-month RMI**	N/A		-5,077 (-6,996 to -3,159)	<0.001	N/A		N/A	
**3-month BI**	N/A		N/A		-3,641 (-5,147 to -2,136)	<0.001	N/A	
	n	816	n	498	n	532	n	843

eTable 13. Generalized linear models for the association of functional improvement per the modified Rankin Scale (mRS), Rivermead Mobility Index (RMI), and/or Barthel Index (BI) between 3-months to 1-year post-stroke, with 5-year health and social care costs, adjusted for age, sex, stroke subtype (lacunar vs non-lacunar), 3-month score on the relevant measure, and recurrent strokes during follow-up in 1-year survivors of ischaemic stroke, excluding those with pre-stroke disability (mRS>2, and also BI<20 for analyses with RMI/BI). Each model is further adjusted for additional characteristics differing significantly between patients who did or did not demonstrate late improvement per that parameter (hazards not shown): for mRS, this included prior TIAs and angina; for BI, this included dyslipidemia, peripheral vascular disease, initial NIHSS, and inpatient rehabilitation. The models also exclude patients without full 5-years follow-up (n=173), those already living in a care home pre-stroke (n=30), and those who could not show improvement by definition for each model.

	mRS improvement between 3-12 months		RMI improvement between 3-12 months		BI and/or RMI improvement 3-12m		mRS, RMI, and/or BI improvement 3-12m	
	Margin,$ (95%CI)	p>|z|	Margin, $	p>|z|	Margin, $	p>|z|	Margin, $	p>|z|
**Late functional improvement**	-16,439 (-24,467 to -8,411)	<0.001	-12,240 (-22,228 to -2,252)	0.016	-13,490 (-23,440 to -3,541)	0.008	-8,143 (-14,271 to -2,014)	0.009
**Age**	725 (437-1,013)	<0.001	538 (117-958)	0.012	695 (275-1,115)	0.001	722 (454-990)	<0.001
**Male sex**	-3,173 (-9,157-2,811)	0.30	2,207 (-7,054-11,468)	0.64	-3,218 (-12,307-5,871)	0.49	-4,263 (-9,986-1,461)	0.14
**Lacunar stroke**	-5,262 (-12,326-1,802)	0.14	-8,913 (-20,326-2,500)	0.13	-9,831 (-21,309-1,647)	0.093	-5,982 (-12,734-770)	0.082
**3-month mRS**	1=Reference		N/A		N/A			
**2**	3,499 (-415-7,413)	0.080					6,824 (1,564-12,084)	0.011
**3**	18,383 (9,304-27,463)	<0.001					22,822 (12,818-32,836)	<0.001
**4**	54,747 (28,120-81,373)	<0.001					54,663 (29,595-79,732)	<0.001
**5**	96,067 (35,237-156,898)	0.002					97,373 (38,069-156,677)	0.001
**3-month RMI**	N/A		-4,704 (-6,689 to -2,718)	<0.001	N/A		N/A	
**3-month BI**	N/A		N/A		-4,235 (-6,520 to -1,950)	<0.001	N/A	
**Recurrent stroke**	16,838 (7,840-25,835)	<0.001	23,956 (9,640-38,271)	0.001	25,968 (11,637-40,299)	<0.001	17,047 (8,355-25,739)	<0.001
	n	833	n	427	n	481	n	866

eTable 14. Models for the association of functional improvement per the modified Rankin Scale (mRS) between 3-months to 1-year post-stroke, with 5-year mortality, institutionalization, mortality/institutionalization,(Cox regression) and 5-year health/social-care costs (generalized linear model), adjusted for age, sex, stroke subtype (lacunar vs non-lacunar), and 3-month score on the relevant measure, in 1-year survivors of ischaemic stroke with 3-month mRS of 2-4. The model for 5-year mortality is further adjusted for recurrent events during follow-up. Models examining institutionalization, mortality/institutionalization, and health/social-care costs exclude these patients as well as those who were already living in a nursing or residential care home pre-stroke, and the model examining costs further excludes patients without full five years of follow-up (n=60).

	**5-year mortality**		**5-year institutionalization**		**5-year mortality/institutionalization**		**5-year health/social-care costs**	
	**aHR(95%CI)**	**p>|z|**	**aHR(95%CI)**	**p>|z|**	**aHR(95%CI)**	**p>|z|**	**Margin, $ (95%CI)**	**p>|z|**
**Late functional improvement**	0.71 (0.51-0.98)	0.038	0.50 (0.30-0.85)	0.011	0.67 (0.48-0.92)	0.014	-20,306 (-30,211 to -10,402)	<0.001
**Age**	1.8 (1.06-1.10)	<0.001	1.08 (1.05-1.11)	<0.001	1.09 (1.07-1.11)	<0.001	612 (216-1,008)	0.002
**Male sex**	1.34 (1.01-1.78)	0.045	1.01 (0.66-1.54)	0.96	1.23 (0.94-1.61)	0.14	-4,199 (-12,439-4,041)	0.32
**Lacunar stroke**	0.79 (0.52-1.19)	0.26	0.77 (0.42-1.42)	0.40	0.67 (0.45-1.01)	0.056	-4,801 (-15,226-5,625)	0.37
**3-month mRS**	2=Reference		2=Reference		2=Reference		2=Reference	
**3**	1.72 (1.25-2.37)	0.001	3.98 (2.14-7.42)	<0.001	2.22 (1.60-3.07)	<0.001	18,182 (10,428-25,937)	<0.001
**4**	1.72 (1.18-2.51)	0.004	8.25 (4.39-15.5)	<0.001	2.85 (1.98-4.11)	<0.001	43,058 (26,050-60,067)	<0.001
	$p>\|X^2\|$	<0.0001	$p>\|X^2\|$	<0.0001	$p>\|X^2\|$	<0.0001	$p>\|X^2\|$	<0.0001
	n	638	n	529	n	544	n	473

eTable 15. Cox regression models for the association of functional improvement per the modified Rankin Scale (mRS), Rivermead Mobility Index (RMI), and/or Barthel Index (BI) between 3-months to 1-year post-stroke, coded as numerical variables, with 5-year mortality/institutionalization, adjusted for age, sex, stroke subtype (lacunar vs non-lacunar), 3-month score on the relevant measure, and recurrent strokes during follow-up, in 1-year survivors of ischaemic stroke. Models exclude those patients who were already living in a nursing or residential care home pre-stroke (n=30).

	Hazard for 5-year mortality/institutionalization					
	mRS improvement between 3-12 months		RMI improvement between 3-12 months		BI improvement between 3-12 months	
	aHR(95%CI)	p>|z|	aHR(95%CI)	p>|z|	aHR(95%CI)	p>|z|
**Late functional improvement**	0.63 (0.51-0.79)	<0.001	0.85 (0.79-0.91)	<0.001	0.91 (0.86-0.95)	<0.001
**Age**	1.08 (1.07-1.09)	<0.001	1.07 (1.05-1.09)	<0.001	1.07 (1.05-1.09)	<0.001
**Male sex**	1.14 (0.93-1.40)	0.22	1.26 (0.99-1.62)	0.065	1.23 (0.94-1.60)	0.14
**Lacunar stroke**	0.68 (0.51-0.92)	0.011	0.64 (0.45-0.92)	0.014	0.59 (0.40-0.88)	0.010
**3-month mRS**			N/A		N/A	
**2**	1.34 (0.96-1.86)	0.084				
**3**	2.93 (2.16-3.96)	<0.001				
**4**	4.10 (2.94-5.72)	<0.001				
**5**	9.37 (6.38-13.8)	<0.001				
**3-month RMI**	N/A		0.86 (0.84-0.89)	<0.001	N/A	
**3-month BI**	N/A		N/A		0.89 (0.87-0.91)	<0.001
**Recurrent Stroke**	1.58 (1.24-2.03)	<0.001	1.47 (1.10-1.96)	0.009	1.61 (1.16-2.22)	0.004
	$p>\|X^2\|$	<0.0001	$p>\|X^2\|$	<0.0001	$p>\|X^2\|$	<0.0001
	n	1,135	n	706	n	450

eTable 16. Generalized linear models for the association of functional improvement per the modified Rankin Scale (mRS), Rivermead Mobility Index (RMI), or Barthel Index (BI) between 3-months to 1-year post-stroke, coded as numerical variables, with 5-year health/social-care costs, adjusted for age, sex, stroke subtype (lacunar vs non-lacunar), 3-month score on the relevant measure, and recurrent strokes during follow-up, in 1-year survivors of ischaemic stroke. Models exclude those patients who were already living in a nursing or residential care home pre-stroke (n=30), as well as patients without full five years of follow-up (n=60).

	mRS improvement between 3-12 months		RMI improvement between 3-12 months		BI improvement between 3-12 months	
	**Margin,$ (95%CI)**	**p>|z|**	**Margin, $**	**p>|z|**	**Margin, $**	**p>|z|**
**Late functional improvement**	-12,938 (-18,998 to -6,877)	<0.001	-5,603 (-8,709 to -2,498)	<0.001	-4,016 (-6,830 to -1,202)	0.005
**Age**	680 (409-951)	<0.001	718 (283-1,153)	0.001	1,035 (376-1,694)	0.002
**Male sex**	-3,896 (-9,711-1,919)	0.19	835 (-8,268-9,938)	0.86	-7,297 (-20,004-5,410)	0.26
**Lacunar stroke**	-6,251 (-13,615-1,114)	0.096	-11,035 (-22,487-417)	0.059	-11,917 (-28,678-4,843)	0.16
**3-month mRS**	1=Reference		N/A		N/A	
**2**	3,821 (61.6-7,580)	0.046				
**3**	20,857 (13,365-28,348)	<0.001				
**4**	42,429 (27,528-57,329)	<0.001				
**5**	67,648 (36,186-99,111)	<0.001				
**3-month RMI**	N/A		-5,897 (-7,964 to -3,830)	<0.001	N/A	
**3-month BI**	N/A		N/A		-4,707 (-6,586 to -2,828)	<0.001
**Recurrent stroke**	15,958 (7,544-24,372)	<0.001	20,355 (7,419-33,291)	0.002	18,779 (1,321-36,237)	0.035
	n	1,039	n	606	n	393
